# The role of recombination dynamics in shaping signatures of direct and indirect selection across the *Ficedula* flycatcher genome^†^

**DOI:** 10.1098/rspb.2023.2382

**Published:** 2024-01-17

**Authors:** Madeline A. Chase, Maurine Vilcot, Carina F. Mugal

**Affiliations:** ^1^ Department of Ecology and Genetics, Uppsala University, 75236 Uppsala, Sweden; ^2^ Swiss Ornithological Institute, 6204 Sempach, Switzerland; ^3^ CEFE, University of Montpellier, CNRS, EPHE, IRD, 34293 Montpellier 5, France; ^4^ Laboratory of Biometry and Evolutionary Biology, University of Lyon 1, CNRS UMR 5558, 69622 Villeurbanne cedex, France

**Keywords:** meiotic recombination, linked selection, direct selection, speciation, Hill–Robertson interference

## Abstract

Recombination is a central evolutionary process that reshuffles combinations of alleles along chromosomes, and consequently is expected to influence the efficacy of direct selection via Hill–Robertson interference. Additionally, the indirect effects of selection on neutral genetic diversity are expected to show a negative relationship with recombination rate, as background selection and genetic hitchhiking are stronger when recombination rate is low. However, owing to the limited availability of recombination rate estimates across divergent species, the impact of evolutionary changes in recombination rate on genomic signatures of selection remains largely unexplored. To address this question, we estimate recombination rate in two *Ficedula* flycatcher species, the taiga flycatcher (*Ficedula albicilla*) and collared flycatcher (*Ficedula albicollis*). We show that recombination rate is strongly correlated with signatures of indirect selection, and that evolutionary changes in recombination rate between species have observable impacts on this relationship. Conversely, signatures of direct selection on coding sequences show little to no relationship with recombination rate, even when restricted to genes where recombination rate is conserved between species. Thus, using measures of indirect and direct selection that bridge micro- and macro-evolutionary timescales, we demonstrate that the role of recombination rate and its dynamics varies for different signatures of selection.

## 1. Introduction

Meiotic recombination is a central evolutionary process with many effects on genome evolution. Recombination can create novel combinations of alleles that aid adaptation, which is hypothesized to contribute to the origin of sexual reproduction [2,3]. However, recombination may also be a disadvantage by breaking apart existing adaptive associations. The suppression of recombination can therefore occasionally promote local adaptation and speciation [4,5], for instance when inversions capture multiple loci with beneficial variation. Besides breaking up linkage among sites, recombination is also associated with the process of GC-biased gene conversion (gBGC), which leads to the preferential fixation of G : C over A : T alleles and can interfere with fitness [6,7]. Thus, recombination can have a multi-faceted impact on fitness.

**Figure 1.**
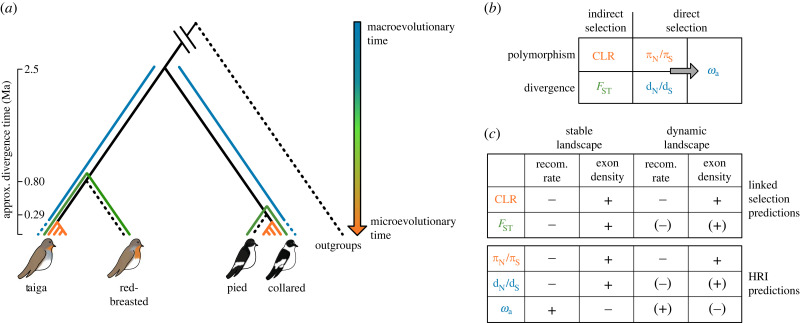
Outline of the study design and working hypothesis. (*a*) Topology of the species included in the study, with approximate divergence times based on demographic modelling. Species that were used as outgroups or as reference species for estimating pairwise *F*_ST_ are represented by dashed black lines. To the right of the topology we indicate the timescale in colour, from a macroevolutionary (blue) to a microevolutionary (orange) timescale. The corresponding colour code is used for the measures of natural selection that we estimate (*b*). (*c*) Summary of the predicted relationships between different measures of selection and recombination rate and density of selected sites in the presence of linked selection and Hill–Robertson interference (HRI). Predictions are presented for a stable recombination landscape versus a dynamic recombination landscape, where correlations are shown in parentheses if we expect them to be weakened by a dynamic recombination rate.

Variation in recombination rate across the genome can also play a major role in shaping genomic signatures of natural selection. Recombination rate can impact the efficacy of selection via Hill–Robertson interference (HRI), where linkage between multiple non-neutral mutations causes selective interference [8]. HRI predicts that the efficacy of natural selection should increase with increasing recombination rate, reflected by an increased fixation of deleterious mutations where recombination rate is low and increased fixation of beneficial mutations where recombination rate is high. Typical measures used to assess the presence of HRI are the nonsynonymous over synonymous ratios of diversity *π*_N_/*π*_S_ and divergence *d*_N_/*d*_S_, which are predicted to show a negative relationship with recombination rate, and the adaptive substitution rate *ω*_a_, predicted to show a positive relationship with recombination rate (figure 1*c*). Physical linkage among sites also manifests in a reduction of genetic diversity at neutral sites that are linked to targets of selection. The process by which neutral diversity is reduced through physical linkage to a beneficial mutation was originally referred to as genetic hitchhiking (HH) [9], leaving the signature of a selective sweep [9–11]. The size of a selective sweep depends on both the strength of selection and the recombination rate, with the reduction in diversity rapidly decreasing with increasing recombination [12,13]. This means selective sweep signatures are predicted to correlate negatively with recombination rate (figure 1*c*). Neutral diversity can also be reduced at sites linked to novel deleterious mutations by background selection (BGS), with a greater impact where recombination rate is lower [14]. We will jointly refer to the impacts of selective sweeps and BGS as indirect selection, to emphasize the feature that they refer to the indirect effects of selection on linked, neutral sites. Although selective sweeps and BGS leave several similar genomic signatures, there are key features that allow the distinction of, in particular, hard selective sweeps from BGS, such as the derived allele frequency spectrum [10]. Nevertheless, the diversity reducing effects of both types of indirect selection can lead to increased genetic differentiation (*F*_ST_) between species [15,16], resulting in a negative relationship between *F*_ST_ and recombination rate (figure 1*c*).

Many studies have investigated the relationship between recombination rate and genomic signatures of indirect selection. Across a wide range of taxa, variation in genetic diversity is strongly correlated with recombination rate [17–19]. Differentiation islands, genomic regions showing significantly elevated *F*_ST_ relative to the genomic background, frequently correspond to regions of reduced recombination [20–23]. Reproductive barrier loci and reductions in introgression have also been observed in regions of low recombination [24–27]. Altogether, these observations point to a pervasive role of recombination rate shaping signatures of indirect selection and correspond well with the expectations from population genetic theory.

On the other hand, evidence for recombination rate shaping signatures of direct selection via HRI appears less consistent across different taxa. HRI has been suggested to play a role in shaping patterns of molecular evolution in many systems, including, among others, the invertebrates *Drosophila melanogaster* [28,29], *Drosophila pseudoobscura* [30], *Heliconius melpomene* [31], the vertebrate great tit *Parus major* [32] and the plant sorrel (*Rumex hastatulus*) [33]. However, in other systems, including collared flycatcher (*Ficedula albicollis*), there has been no evidence of HRI shaping the efficacy of selection across the genome [34–37]. The intensity of HRI relies on multiple interacting variables, which can differ greatly among divergent taxa and could explain the mixed evidence for a relationship between recombination rate and signatures of direct selection. Both the density of functional sites and mutation rate contribute to the strength of HRI because interference requires multiple selected mutations to be linked and segregating at the same time. Neutrally evolving sites occurring in between selected sites (i.e. introns in between exons) can lessen the impact of interference by increasing the probability of recombination between interfering sites [38,39]. Thus, the impact of HRI on measures of direct selection may be more apparent in more compact genomes, such as invertebrate genomes, where gene density is higher compared with vertebrates and plants. Additionally, a relationship between recombination rate and genomic signatures of direct selection may not have the chance to build up if the recombination landscape evolves rapidly [30].

The dynamics of recombination rate evolution are highly variable across different taxa. In many mammals, a rapid turnover of recombination hotspots is observed. The fast evolution of hotspots is mediated by the zinc-finger protein PRDM9 [40–42], which encodes the location of hotspots and is rapidly evolving in response to the erosion of hotspot motifs [43–45]. In organisms that lack PRDM9, such as birds, recombination hotspots tend to be more stable over evolutionary time and the recombination landscape evolves at a slower pace [46,47]. Broad-scale variation in recombination rate also changes over time, for example, owing to chromosomal rearrangements [48–50]. However, despite a broad acknowledgement that recombination rate is dynamic, only few studies have directly investigated the impact of recombination rate evolution on genomic signatures of natural selection. Given the clear importance of recombination rate in shaping genomic signatures of selection, more investigation is warranted into how the evolutionary dynamics of recombination rate among species impacts these genomic signatures.

Here we investigate the role of recombination rate dynamics in shaping patterns of indirect and direct selection in two species of *Ficedula* flycatchers (figure 1*a*), taiga flycatcher and collared flycatcher. These two species diverged at an estimated 2.5 Ma, share a negligible amount of polymorphisms (approx. 2%), and show no recent history of gene flow [51], and both have a more closely related sister species, red-breasted flycatcher and pied flycatcher, respectively, for which population re-sequencing data are available to compute *F*_ST_ landscapes (figure 1*a*). Therefore, these two species constitute an excellent study system to infer evolutionary changes in local recombination rate in birds and to assess their impact on genomic signatures of selection. Specifically, we estimate lineage-specific recombination rates in taiga flycatcher and collared flycatcher, as well as four measures of selection, with two measures each representing genomic signatures of indirect and direct selection, using both polymorphism-based and divergence-based measures, which reflect different timescales (figure 1). These measures include the composite likelihood ratio (CLR) test for selective sweeps, genetic differentiation *F*_ST_, which in *Ficedula* flycatchers is suggested to be governed by linked selection [20,51], *π*_N_/*π*_S_, and *d*_N_/*d*_S_ (figure 1*b*). Additionally, we estimate the adaptive substitution rate (*ω*_a_), which relies on a combination of polymorphism and divergence data. We then investigate the relationship between evolutionary changes in recombination rate and these measures of selection. We also study the association between selective sweeps and *ω*_a_, i.e. two distinct genomic signatures of positive selection, which respectively assess indirect and direct selection. In brief, we address the following questions: (1) How does recombination rate impact genomic signatures of indirect and direct selection in *Ficedula* flycatchers? (2) How do the dynamics of recombination rate evolution impact both signatures of selection? and (3) How do signatures of indirect and direct selection compare with one another?

## 2. Results

### (a) Study system and whole genome re-sequencing data

To understand the relationship between the evolutionary dynamics of recombination rate and genomic signatures of indirect and direct selection, we collated whole genome re-sequencing data for four species of *Ficedula* flycatchers: 65 taiga flycatchers (*Ficedula albicilla*) [51], 15 red-breasted flycatchers (*Ficedula parva*) [51], 95 collared flycatchers (*Ficedula albicollis*) [52] and 11 pied flycatchers (*Ficedula hypoleuca*) [20]. Additionally, we included one individual of snowy-browed flycatcher (*Ficedula hyperythra*) [20] as an outgroup for variant polarization. We performed variant calling on all five flycatcher species and identified in total 51 424 863 single nucleotide variants (SNVs) within a set of 566 724 393 callable sites. We then focused our study on the more distant comparison of taiga flycatcher and collared flycatcher, while red-breasted flycatcher and pied flycatcher are only included for SNV polarization and to estimate pairwise *F*_ST_ (figure 1).

To assess how the demographic history of our study system might influence our results, we performed demographic modelling of the divergence between the two closely related pairs of species using the software GADMA [53] and reconstructed species-specific demographic history using PSMC [54]. Recombination rate estimates based on linkage disequilibrium (LD) have been shown to be influenced by recent demography, where fine-scale variation and recombination hotspot detection appear to be sensitive to population bottlenecks [55], while broad-scale estimates of recombination rate may be more robust to demography [56]. Recent work also suggests that gene flow between species could confound genome-wide estimates of recombination rate from LD-based methods [57]. Although the best fitting model identified by GADMA included gene flow for both species pairs (electronic supplementary material, table S1 and figure S1), the effective migration rates estimated here appear sufficiently low as not to show an impact on LD-based recombination estimates [57]. However, owing to the sensitivity of fine-scale recombination rate on demography, in this study we focus our analysis on broad-scale variation in recombination rate across the genome, at a resolution of 200 kb windows, which also enables us to validate our findings based on LD-based recombination rate estimates by pedigree-based recombination rate estimates for collared flycatcher [58].

### (b) The evolutionary dynamics of recombination rate among taiga and collared flycatchers

**Figure 2.**
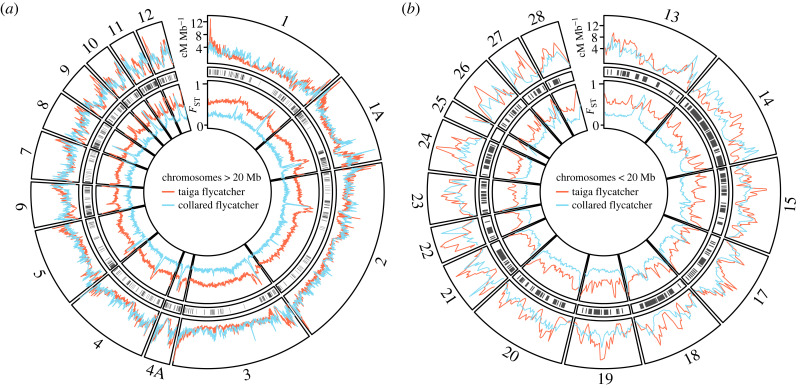
Recombination and differentiation landscapes. (*a*,*b*) LD-based recombination landscapes across the genome for the taiga flycatcher (red) and collared flycatcher (blue), and genome-wide variation in *F*_ST_ between taiga and red-breasted flycatcher (red) and collared and pied flycatcher (blue). Grey rectangles display regions where recombination rate is divergent between taiga flycatcher and collared flycatcher.

To assess the evolutionary dynamics of recombination rate in *Ficedula* flycatchers, we estimated recombination rate in taiga flycatcher and collared flycatcher based on patterns of LD. LD-based estimates for both taiga flycatcher and collared flycatcher showed significant correlations with pedigree-based estimates for collared flycatcher at 200 kb resolution [58], with Pearson's correlation coefficients *R* = 0.46 and *R* = 0.58, *p*-value < 10^−16^, respectively (electronic supplementary material, figure S2). The strength of the correlation increased with increasing window size and was highest in 5 Mb windows (*R* = 0.77 and *R* = 0.76*, p-*value < 10^−16^ for taiga flycatcher and collared flycatcher; electronic supplementary material, figure S3). These observations and the strength of the correlations are in good agreement with observations based on pedigree maps in other organisms [59,60], where it has been shown that the resolution of pedigree-based estimates can limit the strength of the correlation with LD-based estimates of recombination rate [60,61]. We next converted LD-based recombination rate from population-scaled recombination (*ρ* = 4*N_e_r*) into cM Mb^−1^ (figure 2*a,b*) using the linkage map.

We observed a significant correlation for recombination rate between the two species estimated in 200 kb windows (electronic supplementary material, figure S4; Pearson's *R* = 0.45, *p*-value < 10^−16^), which was weaker than correlations observed previously between the more closely related collared and pied flycatchers (Pearson's *R* = 0.79) [47]. We identified that 24% of 200 kb windows showed differences in the recombination rate between the two species, and classified windows into windows with conserved and divergent recombination rate (figure 2*a,b*). To investigate if the inferred changes in recombination rate represent an evolutionary signal or mainly reflect uncertainty in LD-based recombination rate estimates, we compared the correlation between LD-based recombination rate of both species and pedigree-based recombination rate of collared flycatcher separately for the conserved and divergent windows. We expect an evolutionary signal to manifest in a lower correlation for divergent compared with conserved windows for LD-based recombination rate of taiga flycatcher but not collared flycatcher. By contrast, if a low signal-to-noise ratio explains divergent windows, then a lower correlation with the pedigree-based estimates is expected for both species in these regions. We observed that collared flycatcher showed similar patterns for both window types (Pearson's *R* = 0.59 and *R* = 0.53 in conserved and divergent windows respectively; electronic supplementary material, figure S5), but for taiga flycatcher the correlation coefficient was reduced by half in divergent windows compared with conserved windows (Pearson's *R* = 0.57 and *R* = 0.24 in conserved and divergent windows respectively; electronic supplementary material, figure S5). Thus, the correlation analysis provides support that inferred evolutionary changes in recombination rate represent an evolutionary signal.

As an additional assessment, we compared signatures of gBGC between the two species separately for conserved and divergent windows. The strength of gBGC should positively correlate with recombination rate [62]; therefore we would expect divergent windows to show a greater impact of gBGC for the species with the higher recombination rate. Our analysis revealed that, relative to conserved regions, divergent regions with higher recombination rate in collared flycatcher showed a greater impact of gBGC in collared flycatcher than in taiga flycatcher, and *vice versa* for divergent regions with higher recombination rate in taiga flycatcher (electronic supplementary material, figure S6). Because inferred signatures of gBGC rely on patterns in the site frequency spectrum (SFS) rather than patterns of LD, these observations provide an additional and independent validation that the inferred changes in recombination rate reflect an evolutionary signal rather than noise in LD-based measurements.

### (c) Recombination rate shapes genomic signatures of indirect selection

**Figure 3.**
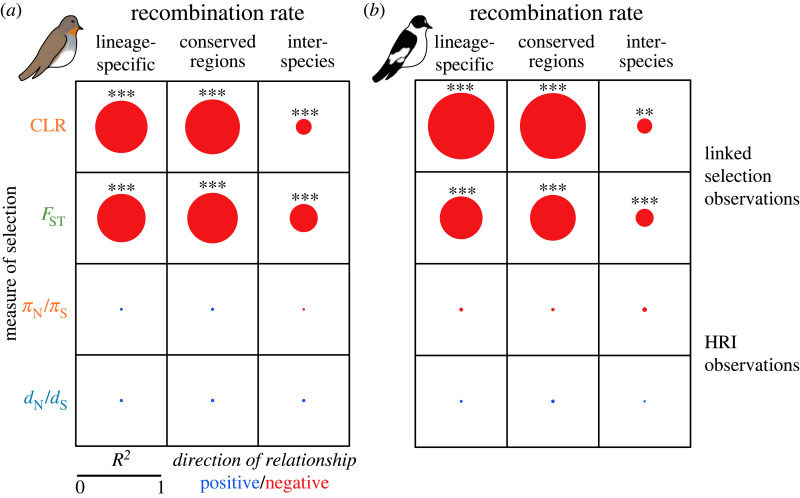
Summary of relationships between genomic signatures of selection and recombination rate. Shown are the *R*^2^ values for four different selection measures and recombination rate. The diameter of the circles indicates the strength of the relationship and the colour represents the direction of the relationship, positive in blue and negative in red. For both species, three comparisons between recombination rate and signatures of selection are shown: lineage-specific recombination rate estimates across all windows, lineage-specific recombination rate estimates across windows with conserved recombination rate, and an interspecies comparison. Asterisks denote significance of the relationship, with *p-value < 0.05, **p-value < 0.01, and ***p-value < 0.001. HRI, Hill–Robertson interference.

We examined the relationship between recombination rate and measures of indirect selection based on two statistics, the CLR test for selective sweeps and pairwise *F*_ST_ between the species pairs taiga and red-breasted flycatcher and collared and pied flycatcher (figure 1*a*). Logistic regression analysis between lineage-specific recombination rate and CLR estimates in both taiga flycatcher and collared flycatcher supported the predicted relationship between recombination and selective sweep signatures (figure 3; electronic supplementary material, table S2 and figure S7), with signatures of selective sweeps tending to occur in windows with lower recombination rate. To investigate how evolutionary changes in recombination rate impact genomic signatures of indirect selection, we also examined correlations across species, which revealed a weaker relationship between selective sweeps estimated in one species and recombination rate estimated in the other (figure 3; electronic supplementary material, table S2 and figure S7).

Next, we compared how lineage-specific estimates of recombination rate corresponded with the differentiation landscape for the two species comparisons. We observed a negative relationship between recombination rate and *F*_ST_ for both species pairs, taiga and red-breasted flycatchers (figure 3; electronic supplementary material, figure S8; Spearman's *R* = −0.71; *p*-value < 2.2 × 10^−16^) and collared and pied flycatchers (figure 3; electronic supplementary material, figure S8; Spearman's *R* = −0.59; *p*-value < 2.2 × 10^−16^), which is in good agreement with the linked-selection prediction. As with the selective sweep analysis, the relationship between recombination rate and *F*_ST_ was lower for interspecies comparisons (figure 3; electronic supplementary material, figure S8), and slightly higher when restricted to conserved recombination rate regions (figure 3). We also identified *F*_ST_ peaks between both species pairs, where local *F*_ST_ was significantly higher than the chromosomal background, which we then categorized as lineage-specific or shared between the species comparisons (electronic supplementary material, table S3). Both taiga flycatcher and collared flycatcher showed significantly reduced recombination rate in shared *F*_ST_ peaks (electronic supplementary material, figure S9) compared with windows without *F*_ST_ peaks. Collared flycatcher showed significantly lower recombination rate in collared/pied-specific (CP unique) *F*_ST_ peaks, but not in taiga/red-breasted-specific (TR unique) peaks (electronic supplementary material, figure S9). Although taiga flycatcher showed a significant reduction in recombination rate for both TR unique peaks as well as CP unique peaks compared with the genomic background, the reduction was greater for shared and TR unique peaks compared with CP unique peaks (electronic supplementary material, figure S9). Overall, these results support that evolutionary changes in recombination rate shape lineage-specific signals of *F*_ST_ peaks. Note also that for collared flycatcher, variation in LD-based recombination rate among *F*_ST_ peaks recaptures earlier observations using the pedigree-based recombination rate ([51]; electronic supplementary material, figure S6*b*). Thus, our results suggest that neglecting evolutionary changes in recombination rate can provide a biased picture of the action of natural selection.

Previous studies have demonstrated that recombination rate is often reduced towards the centres of chromosomes [47,63], an observation replicated here for the macrochromosomes (figure 2; electronic supplementary material, figure S10), while the opposite pattern was observed on microchromosomes (figure 2; electronic supplementary material, figure S10). We investigated whether there was also an impact of the relative location along the chromosome on the presence of *F*_ST_ peaks. For both species, *F*_ST_ peaks were enriched in the ends of microchromosomes, where recombination rate was reduced, suggesting that chromosomal location does impact *F*_ST_ peak prevalence (electronic supplementary material, figure S10). In macrochromosomes there was little variation among chromosomal regions for collared flycatcher (electronic supplementary material, figure S10); however, *F*_ST_ peaks were more prevalent in chromosome ends for taiga flycatcher (electronic supplementary material, figure S10). It is therefore possible that chromosomal rearrangements that change the relative location of regions along the chromosome may contribute to generating some of the observed changes in recombination rate and associated lineage-specific *F*_ST_ peaks.

### (d) Genomic signatures of direct selection are not consistent with HRI

To assess evidence for HRI in taiga flycatcher and collared flycatcher, we compared the relationship between recombination and two lineage-specific signatures of direct selection, *π*_N_/*π*_S_ and *d*_N_/*d*_S_, on a gene-by-gene basis. To account for the noisiness of gene-based estimates of *π*_N_/*π*_S_ and *d*_N_/*d*_S_, we also assessed evidence for HRI using a binning approach. For this purpose, we assigned genes to one of three bins with varying recombination rate, after estimating the per gene average recombination rate (electronic supplementary material, table S4). The binning approach permits estimation of the distribution of fitness effects (DFE), particularly the mean strength of selection against deleterious mutations *N*_e_*s*, and *ω*_a_, for which there is insufficient power on a gene-by-gene basis. Since we might expect that any relationship between recombination rate and measures of direct selection would be weakened by evolutionary changes in recombination rate, we additionally estimated *π*_N_/*π*_S_, *d*_N_/*d*_S_, and *ω*_a_ for the subset of genes with conserved gene-based recombination rate between taiga flycatcher and collared flycatcher. We also examined whether variation in the density of functional sites might obscure patterns of HRI, by dividing recombination rate bins into low and high functional density (i.e. density of exons and conserved non-coding elements (CNEs); electronic supplementary material, table S5). Finally, to account for any potential bias due to gBGC, estimates of *π*_N_/*π*_S_, *N*_e_*s*, *d*_N_/*d*_S_ and *ω*_a_ are based on GC-conservative changes only.

Based on the gene-by-gene analysis as well as the binning approach we did not observe the relationships between recombination rate and signatures of direct selection predicted by HRI in either species, regardless of whether we restricted the analysis to genes with conserved recombination rate or not (figure 3; electronic supplementary material, figures S11 and S12). Accounting for variation in functional density, we again observed no consistent pattern between species that matched the predictions based on HRI for recombination rate and signatures of direct selection (electronic supplementary material, figures S13 and S14). Taken together, our results therefore suggest a negligible role of recombination in shaping genomic signatures of direct selection in *Ficedula* flycatchers.

### (e) Weak association between signatures of direct and indirect selection

Finally, we compared genomic signatures of direct and indirect selection with each other to address the hypothesis that recurrent selective sweeps may contribute to *F*_ST_ peaks, reflected by an increased rate of adaptive substitutions in *F*_ST_ peaks compared with the genomic background. For this purpose, we compared estimates of *π*_N_/*π*_S_, *N*_e_*s*, *d*_N_/*d*_S_ and *ω*_a_ for genes overlapping with shared *F*_ST_ peaks, lineage-specific *F*_ST_ peaks, and *F*_ST_ peaks with or without a selective sweep signature against genes not overlapping with *F*_ST_ peaks as a background reference. Lineage-specific *F*_ST_ peaks and *F*_ST_ peaks without a selective sweep signature showed no significant differences in signatures of direct selection compared with the genomic background. Both taiga flycatcher and collared flycatcher showed significantly higher π_N_/*π*_S_ in shared *F*_ST_ peaks relative to the genomic background (table 1), and taiga flycatcher showed significantly larger *d*_N_/*d*_S_ and *ω*_a_. For collared flycatcher, estimates of *d*_N_/*d*_S_ were elevated in shared *F*_ST_ peaks but differences were not statistically significant. Similarly, *π*_N_/*π*_S_ was significantly higher for both species within *F*_ST_ peaks overlapping with selective sweeps, while differences in *d*_N_/*d*_S_ were only significantly larger for taiga flycatcher, and differences in *ω*_a_ were not statistically significant for either species (table 1).

**Table 1.** Comparison of measures of indirect and direct selection. Shown are estimates of the efficacy of direct selection for genes grouped by *F*_ST_ peak category. Categories include genes outside *F*_ST_ peaks, genes in *F*_ST_ peaks shared between species comparisons, genes within peaks specific to the focal species, genes within peaks that show a signature of a selective sweep, and genes within peaks that do not show a signature of a selective sweep. *p*-values comparing whether statistics within *F*_ST_ peaks are significantly different from outside *F*_ST_ peaks are presented in parentheses. Significant differences are indicated in bold, *p*-value < 0.05. See electronic supplementary material, table S6 for estimates based on all changes.

	taiga flycatcher					collared flycatcher				
	*π*_N_/*π*_S_	*N*_e_*s*	*d*_N_/*d*_S_	*ω*_a_	genes	*π*_N_/*π*_S_	*N*_e_*s*	*d*_N_/*d*_S_	*ω*_a_	genes
outside peaks	0.17	1485	0.19	0.087	5862	0.17	34852	0.18	0.029	5842
shared peaks	**0.34** **(0.001)**	146 (0.16)	**0.32** **(0.021)**	**0.21** **(0.034)**	245	**0.32** **(0.002)**	4789 (0.70)	0.23 (0.25)	−0.00084 (0.65)	241
lineage-specific peaks	0.12 (0.15)	149 (0.24)	0.17 (0.79)	0.14 (0.37)	172	0.086 (0.16)	208 (0.53)	0.091 (0.29)	0.063 (0.71)	82
peaks with sweep overlap	**0.46** **(0.001)**	111 (0.21)	**0.31** **(0.038)**	0.15 (0.26)	201	**0.53** **(0.006)**	8.3 (0.67)	0.093 (0.43)	**−0.37** **(0.041)**	52
peaks with no sweep overlap	0.20 (0.39)	190 (0.21)	0.21 (0.72)	0.13 (0.39)	206	0.23 (0.10)	13818 (0.84)	0.24 (0.22)	0.073 (0.45)	265

## 3. Discussion

### (a) Signatures of indirect selection are shaped by recombination rate dynamics

Speciation genomic studies across a wide range of species have revealed that differentiation islands tend to coincide with regions of low recombination as a consequence of a local reduction of genetic diversity owing to indirect selection [20–22,26,51]. Despite the central role of recombination in shaping the differentiation landscape, the role of the evolutionary dynamics of recombination rate in these patterns has largely remained unexplored, often because recombination rate estimates have only been available for one of the studied species, or not available for the focal species ([20,22,26,51] but see [47]). Recent work has, however, demonstrated that not accounting for recombination rate evolution can lead to an underestimation of the impact of recombination rate on signatures of indirect selection [64], particularly in species where *PRDM9* entails highly dynamic recombination rates, such as mammals. Birds, in contrast, lack *PRDM9* and their genomes are characterized by a more stable fine-scale recombination landscape, which may lead to the conclusion that estimates of recombination rate from one species are sufficient to explain patterns of selection also at the broad-scale. Nevertheless, by estimating recombination rate in two *Ficedula* flycatcher species we provide evidence that evolutionary changes in recombination rate are central in driving differences in signatures of indirect selection between species, while evolutionarily stable reductions in recombination rate manifest in differentiation islands that are shared among species. Therefore, we show that even in species that lack *PRDM9*, accounting for variation in recombination rate between species is crucial to interpreting genomic signatures of natural selection. We thereby expand on our understanding from previous studies where recombination rate estimates were only available for a single species.

Characteristic for the *Ficedula* flycatchers is that both lineage-specific and shared signatures of indirect selection generally stretch several hundred kilobases, suggesting that the mechanism behind low-recombining regions acts at the broad-scale rather than the fine-scale. One possible mechanism for broad-scale reductions in recombination rate that could also generate changes in recombination rate between species involves chromosomal rearrangements such as inversions or translocations [48–50]. Inversions can lead to a reduction in recombination when polymorphic owing to the absence of crossing over in heterozygotes, while translocations can relocate chromosomal segments to a location where recombination rate differs. For example, reduced recombination is often observed in chromosome centres [63], and we observed that chromosomal location was associated with *F*_ST_ peak prevalence, with differences observed between the two species. Aside from chromosomal rearrangements, centromeres are also suggested to contribute to broad-scale *F*_ST_ peaks in flycatchers [20]. Previous work has shown that centromere shifts occur in birds [65], which could also explain some of the changes in recombination rate between taiga flycatcher and collared flycatcher. Nevertheless, in several instances we find multiple *F*_ST_ peaks per chromosome, a signature not expected if *F*_ST_ peaks solely coincide with centromeres but pointing towards another mechanism, such as chromosomal rearrangements. While we can currently only speculate as to the molecular mechanisms behind the recombination landscape dynamics between these species, future research into these hypotheses can benefit from recent advancements in long-read sequencing technology.

Yet, regardless of the molecular mechanism that drives local reductions in recombination rate, differentiation islands or selective sweeps would not arise without the action of selection [9,14–16]. Simulation studies suggest a relationship between the rate of selective sweeps and the adaptive substitution rate, at least in the absence of recombination [66]. Since signatures of selective sweeps rapidly break down with increasing recombination rate [12,13], the predicted relationship is less obvious in recombining genomes. The *Ficedula* flycatcher system allows us to explore this relationship, since the comparison of adaptive substitution rates in lineage-specific and shared *F*_ST_ peaks permits assessing the role of recombination dynamics. With variable recombination rate, we did not observe a relationship between lineage-specific *F*_ST_ peaks and any genomic signatures of direct selection. This may be because there has been no increase in adaptive substitutions in these regions or that any adaptive substitutions occurred too recently to be detected with the methods employed. Shared *F*_ST_ peaks and *F*_ST_ peaks coinciding with selective sweeps showed significantly higher *π*_N_/*π*_S_ for both species, which was driven by a greater reduction in *π*_S_ compared with *π*_N_ as both values were reduced compared with the background. Hitchhiking due to a selective sweep is known to cause a reduction in linked diversity [9], and theory suggests that the reduction in diversity is greater for synonymous sites than nonsynonymous sites [67]. Although estimates of the adaptive substitution rate were significantly elevated in the shared *F*_ST_ peaks compared with the genomic background for taiga flycatcher but not for collared flycatcher, the adaptive substitution rate was not significantly larger in *F*_ST_ peaks coinciding with selective sweeps for either of the two species. These results therefore indicate that differentiation islands and selective sweep signatures may not require significantly elevated rates of adaptation in order to manifest in the genome, and highlight again the central role of recombination dynamics in shaping indirect selection signatures.

### (b) Recombination rate is not a major force in shaping signatures of direct selection

In addition to creating more pronounced signatures of indirect selection, tighter linkage between sites owing to low recombination rate is predicted to increase the impact of HRI. The consequence of this relationship is an increase in the efficacy of natural selection with increasing recombination rate, which has been observed in several systems. However, within both taiga flycatcher and collared flycatcher signatures of direct selection generally showed little variation with recombination rate, and did not reflect the relationships predicted by HRI.

There are several explanations why HRI may be less prevalent in *Ficedula* flycatchers compared with other systems. Selective interference occurs when multiple linked, selected mutations segregate within the population at the same time, and it has been demonstrated that the addition of neutral sites between selected sites may help to alleviate selective interference [39]. We are therefore more likely to observe HRI where there is a greater density of functional sequences relative to the recombination rate. For example, gene density per centimorgan in *D. melanogaster,* where evidence for HRI has been observed, is roughly an order of magnitude larger than in flycatcher [68] (FlyBase release FB2022_04; Ensembl v. 107). Additionally, genetic diversity is much higher in *D. melanogaster* compared with flycatchers [69], meaning multiple selected mutations will be more likely to segregate at the same time. These circumstances could explain why we find no effect of recombination rate on selection efficacy in the *Ficedula* flycatchers, even in the most functionally dense regions of the genome. It is important to note, however, that evidence of HRI in birds is inconclusive [32,34,37], and generalization among birds asks for further investigations.

An absence of HRI in flycatchers is nevertheless in good agreement with earlier observations, which suggest that the intensity of selection on coding sequences is impacted more by functional characteristics such as gene expression patterns than by the genomic background [70]. For instance, recent work found that estimates of *d*_N_/*d*_S_ were highly correlated among distantly related species with divergent genomic backgrounds [71], suggesting that conserved gene functions rather than genomic background shape the variation in *d*_N_/*d*_S_ among genes. To investigate if evolution of the recombination landscape could obscure the expected pattern of HRI in flycatchers, we limited our analysis to genes with evolutionarily conserved recombination rate between taiga flycatcher and collared flycatcher. However, we still observed no clear relationship between recombination rate and signatures of direct selection consistent with HRI. This suggests that recombination rate and its dynamics appear to not play a significant role in shaping any differences in signatures of direct selection between the two birds studied here.

### (c) Conclusions

By comparing recombination dynamics with genomic signatures of indirect and direct selection, we show that these different signatures are not shaped by the same evolutionary processes. While signatures of indirect selection appear to be strongly shaped by the recombination landscape and its dynamics, signatures of direct selection are largely unaffected by the genomic background. Rather, gene function and expression patterns may play a more central role in shaping the efficacy of direct selection on protein-coding sequence [70].

## 4. Material and methods

### (a) Samples and genotyping

Whole genome re-sequencing data for 187 flycatchers were collated from previously published work, including 65 taiga flycatchers [51], 15 red-breasted flycatchers [51], 95 collared flycatchers [52], 11 pied flycatchers [20], and one sample of snowy-browed flycatcher as an outgroup [20]. Base quality score recalibrated BAM files with reads mapped to the collared flycatcher reference genome FicAlb1.5 [58] were obtained for all samples. Genotyping was performed individually with HaplotypeCaller in GATK v.4.1, followed by GenotypeGVCFs with all samples combined specifying the *–all-sites* flag to genotype both variant and invariant sites. We removed indels and variable sites with more than one alternative allele using VCFtools v.0.1.16 [72], resulting in a dataset containing 974 084 046 sites in total, including 116 929 990 SNVs.

Here, we focus only on sites located on the autosomes, excluding unassigned scaffolds, the Z-chromosome, and mitochondria. We followed the filtering methods applied in [50], resulting in a final dataset of 566 724 393 callable sites including 51 424 863 SNVs. We polarized variable sites using snowy-browed flycatcher as an outgroup, and combining taiga and red-breasted flycatcher into one group and collared and pied flycatcher into another group. Whenever any two of the three groups were fixed for the same allele, this allele was considered the ancestral state (electronic supplementary material, methods). This resulted in 564 393 274 callable sites and 49 121 805 polarized SNVs.

### (b) Demographic modelling

We investigated historical fluctuations in population size for the four ingroup flycatcher species using the pairwise sequentially Markovian coalescent (PSMC; [54]) with one individual per species. Sites with a read depth less than 10, sites with more than twice the genome-wide average read depth, and 100 bp blocks with more than 20% missing data were excluded. We used parameters following [73] and performed 100 bootstrap replicates. In addition, we examined the demographic history of the two species pairs with the method of ordinary differential equations using moments software [74] implemented in GADMA [53] with the unfolded joint site frequency spectrum (jSFS) computed from ∂a∂i [75]. We used the following parameters: 100 replicates, *μ* = 4.6 × 10^−9^, *g* = 2, effective length *L* = 564 393 274, and *θ*_0_ = 4 µl = 10.38. The best model was selected according to both log-likelihood and composite likelihood Akaike information criterion (CLAIC). Confidence intervals were calculated for parameter estimates of the best fitting model by dividing each dataset into 1 Mb segments and resampling from these segments for bootstrapping.

### (c) Estimation of recombination rate

Population-scaled recombination rate (*ρ* = 4*N*_e_*r*) was estimated for taiga flycatcher and collared flycatcher. Haplotypes were inferred with SHAPEIT2 [76]. We set genome-wide *ρ* to 0.037 [47], the effective population size to 300 000 [77], the –*states* parameter to 200 and *–window* to 0.5. Recombination rate was estimated with LDhelmet [78] using 25 samples with the least missing data (electronic supplementary material, table S7) and following parameters in [44] (electronic supplementary material, methods). We calculated the average LD recombination rate in 200 kb, 1 Mb, and 5 Mb genomic windows, weighted by the distance between SNP pairs accounting for SNP pairs spanning adjacent windows. Additionally, we summarized the weighted average of recombination rate within each protein-coding gene.

Population-scaled recombination rates were converted into cM Mb^−1^ by dividing by the genome-wide *N_e_,* following [48], using the pedigree recombination map of collared flycatcher, in 200 kb, 1-Mb, and 5 Mb windows [58]. This gave an *N_e_* estimate of 318 000–458 000 for taiga flycatcher, and 30 000–42 500 for the Gotland population of collared flycatcher, where the latter is in line with LD-based *N*_e_ estimates from [52]. We used the value of *N*_e_ estimated from 5 Mb windows, which showed the strongest correlation with the linkage map (electronic supplementary material, figure S3), to convert estimates of recombination rate into cM Mb^−1^. For both the window-based and gene-based recombination rates, we divided regions into ‘conserved’ or ‘divergent’ recombination rate between the two species. We performed a principal component analysis (PCA) using the recombination rate estimates for taiga flycatcher and collared flycatcher and identified regions with conserved recombination rate if they were within one standard deviation from the mean of the second principal component. We validated our inference of changes in recombination rate by comparing the strength of gBGC in regions with conserved and divergent recombination rates between species (electronic supplementary material, methods; [47]).

### (d) Estimation of signatures of indirect selection within and between species

To assess signatures of indirect selection, we performed a selective sweep scan within taiga flycatcher and collared flycatcher. A composite likelihood ratio (CLR) test [79] was used to identify selective sweeps, using polarized SNP data (see §4a) for the two species. We ran SweepFinder2 [80] with a pre-computed background SFS from each species (taiga flycatcher *n* = 65 diploid individuals; collared flycatcher *n* = 95), and a grid of locations for every variant. A significance threshold of 46.25 was taken from previous work [51], based on simulations using the collared flycatcher annotation [81] and recombination map [58] to obtain a threshold that accounts for BGS. We merged adjacent sites with significant CLR values, removed sweeps with only one significant site or with a site density less than 1 kb^−1^, and called presence/absence of selective sweeps in 200 kb windows.

We calculated Weir & Cockerham *F*_ST_ [82] between the species pairs taiga and red-breasted flycatcher and collared and pied flycatcher in 200 kb nonoverlapping windows using VCFtools v.0.1.16 [72]. To identify *F*_ST_ peaks, we *Z*-transformed *F*_ST_ separately for each chromosome to account for differences in mean *F*_ST_ among chromosomes, and applied a Savitzky–Golay smoothing filter to the *ZF*_ST_ values using a polynomial of 3 and a filter length of 7. We identified *F*_ST_ peaks where *ZF*_ST_ was two standard deviations above the chromosome mean, and then classified these as peaks into shared and lineage-specific, i.e. taiga–red-breasted unique (TR unique) or as collared–pied unique (CP unique), based on any overlap between the two species comparisons. To compare recombination rate in different *F*_ST_ peak categories, we performed a permutation test, randomizing the peak category and re-estimating the difference in mean recombination rate among 200 kb windows for 1000 permutations.

### (e) Estimation of signatures of direct selection within and between species

To estimate signatures of direct selection on protein-coding sequences we constructed sequence alignments for one-to-one orthologues between taiga flycatcher, collared flycatcher and zebra finch (*Taeniopygia guttata*). Zebra finch sequences were retrieved from Ensembl v.104 for one-to-one orthologues between collared flycatcher and zebra finch. We reconstructed gene sequences for multiple sample sizes of taiga flycatcher and collared flycatcher, to account for the recent divergence time of the two species (approx. 3.76 coalescent units) [51]; see supplementary material, methods for details. For each gene we combined the sequences for all samples into a single fasta file and aligned the sequences using prank v.170427 [83]. Prior to alignment with prank, we ran clustalw v.2.1 [84] to estimate a guide tree for each gene, which we then provided as input to prank, running prank with the *–once* flag to perform one iteration. We estimated signatures of direct selection in taiga flycatcher and collared flycatcher for one-to-one orthologues between flycatcher and zebra finch. Using fourfold and zerofold degenerate sites extracted from the reconstructed ancestral genome, we calculated *π*_N_/*π*_S_ for taiga flycatcher (*n* = 65) and collared flycatcher (*n* = 95). We subset the variable sites for only GC-conservative variants, i.e. strong-to-strong (C and G) variants or weak-to-weak (A and T) variants, to correct for gBGC.

Using Bio++ v.3.0 [85] we estimated *d*_N_/*d*_S_ for taiga flycatcher and collared flycatcher based on a strand-symmetric L95 model [86] implemented in bppml, and mapped substitutions as implemented in mapnh [87]. This allowed us to separate substitutions into different categories and correct for gBGC by restricting the analysis to GC-conservative changes. We excluded genes with an exon length below 200 bp and summarized *d*_N_/*d*_S_ across genes by dividing the average value of *d*_N_ by the average value of *d*_S_. There was little evidence of sample size dependence in the estimates of *d*_N_/*d*_S_ (electronic supplementary material, tables S8 and S9; electronic supplementary material, methods), suggesting that divergence between taiga flycatcher and collared flycatcher is deep enough for phylogenetic analysis [88]. All further analyses with *d*_N_/*d*_S_ were therefore only based on the 32 haplotype sample size for both taiga flycatcher and collared flycatcher.

Using the fourfold and zerofold site frequency spectra and the results of *d*_N_/*d*_S_, we estimated the adaptive substitution rate (*ω*_a_) with the software DFE-alpha [89,90] for bins of genes (electronic supplementary material, tables S4 and S5). The binning approach is outlined in supplementary material, methods. We used the 2-epoch model of demographic history for all analyses, after confirming this was the best fitting model based on all genes (electronic supplementary material, tables S10 and S11). To test for statistically significant differences in measures of selection between gene bins we performed 1000 permutations, reshuffling the genes among bins. We used the same approach to test for differences in measures estimated for different *F*_ST_ peaks compared with the genomic background.

## Data Availability

Sequencing data for all samples are available at the EMBL-EBI European Nucleotide Archive (ENA; http://www.ebi.ac.uk/ena) with the following accession numbers: PRJEB43825 (taiga and red-breasted flycatchers), PRJEB22864 (collared flycatcher), and PRJEB7359 (pied and snowy-browed flycatchers). VCF files used for analyses and recombination rate data are available on Dryad (https://doi.org/10.5061/dryad.q2bvq83nw) [91]. Scripts used for analysis are available at https://github.com/madeline-chase/flycatcher_recom. Supplementary material is available online [92].
